# Occurrence of Perfluorooctanoic Acid and Perfluorooctane Sulfonate in Milk and Yogurt and Their Risk Assessment

**DOI:** 10.3390/ijerph13101037

**Published:** 2016-10-21

**Authors:** Zhenni Xing, Jianjiang Lu, Zilong Liu, Shanman Li, Gehui Wang, Xiaolong Wang

**Affiliations:** School of Chemistry and Chemical Engineering, Shihezi University, Shihezi 832003, China; xzn_xingzhenni@163.com (Z.X.); liuzilong1999@163.com (Z.L.); lishanman8674@sina.com (S.L.); wang_ge_hui@163.com (G.W.); wxlwxlwxl2013@126.com (X.W.)

**Keywords:** perfluorooctanoic acid, perfluorooctane sulfonate, milk, yogurt, average daily intake, Xinjiang

## Abstract

Although perfluorooctanoic acid (PFOA) and perfluorooctane sulfonate (PFOS) have been identified in milk and dairy products in many regions, knowledge on their occurrence in Xinjiang (China) is rare. This study was conducted to measure the levels of PFOA and PFOS in milk and yogurt from Xinjiang and to investigate the average daily intake (ADI) of these two compounds. PFOA and PFOS levels were analyzed using ultrasonic extraction with methanol and solid-phase extraction followed by liquid chromatography–mass spectrometry. Retail milk and yogurt samples present higher detection rates (39.6% and 48.1%) and mean concentrations (24.5 and 31.8 ng/L) of PFOS than those of PFOA (33.0% and 37.0%; 16.2 and 22.6 ng/L, respectively). For raw milk samples, only PFOS was detected. The differences in the levels of the two compounds between samples from the north and south regions were observed, and northern regions showed higher pollution levels than southern regions. On the basis of the retail milk measurements and consumption data, the ADIs of PFOA and PFOS for Xinjiang adults were calculated to be 0.0211 and 0.0318 ng/kg/day, respectively. Furthermore, the estimated intakes of PFOA and PFOS varied among different groupings (age, area, gender, and race) and increased with increasing age. Relevant hazard ratios were found to be far less than 1.0, and this finding suggested that no imminent health damages were produced by PFOA and PFOS intake via milk and yogurt consumption in the Xinjiang population.

## 1. Introduction

Perfluoroalkyl substances (PFASs) are considered an emerging class of persistent organic pollutants with high thermal and chemical stability, strong bioinertia properties, long-distance transportation ability, and potential accumulation and toxicity. Given the unique hydrophobic and lipophobic properties of PFASs, they are used in a wide range of industrial and consumer applications (stain-resistant coatings for carpets and cloths, cosmetics, pesticides, nonstick cookware, food packaging, and fire-fighting foams) [1,2,3]. The large-scale production and use of PFASs over the past several decades have caused the contamination of the environment, biota or even humans [4,5]. Health concerns arising from PFASs have increased because of their ubiquitous presence and potential hazard effects on human health. Human and animal data suggested that PFASs may disrupt endocrine signaling and alter adipocyte profiles and expression of adipocyte genes [6].

In recent years, information on human exposure to PFASs has been published worldwide [7,8,9]. Perfluorooctanoic acid (PFOA) and perfluorooctane sulfonate (PFOS) are the two most frequently detected chemicals in blood and milk in the general population [10]. Because of their long elimination half-lives in humans (3.8 years for PFOA, 5.4 years for PFOS) [11], exploring the pathways of human exposure to these two compounds is necessary. In contrast to some traditional lipophilic pollutants, such as polychlorinated biphenyls, dioxins or furans that can easily accumulate in lipids, PFOA and PFOS tend to form a strong binding affinity with proteins and concentrate along the food chain [12,13]. Hence, food intake, particularly of food high in protein, is a possible route for general population exposure to PFOA and PFOS [14,15]. Vestergren et al. [14] compared the contribution of various pathways (diet, drinking water, and house dust) for the Swedish population exposure to PFOA and PFOS and concluded that dietary intake is the major contributor. The intake of PFOS and PFOA via diet accounted for 85% and 83%, respectively, of the total average intake, which is 6 to 10 times higher than intake through household dust and drinking water. In addition, an estimate of the overall exposure to PFOS and PFOA for the general adult population was reviewed by Fromme et al. [15], and their results showed that dietary intake is responsible for 96.2% PFOS and 98.6% PFOA of the total daily intake. Meanwhile, several studies have reported the occurrence of PFOS and PFOA in a series of foodstuff, including fish and seafood, beef, eggs, dairy products, beans and beverages, in general, relatively high levels are often found in animal-derived food [2,16,17]. Guerranti et al. [2] discovered that milk and dairy products were the most PFOS-contaminated samples with mean concentrations of 1.35 ± 3.45 ng/g among the analyzed food categories, except for fish and meat. Additionally, milk samples present the highest PFOA content (390 pg/g) aside from fish and seafood [16].

Milk and milk-based products are typical animal-derived food commonly consumed by humans. It is reported that milk intake was positively associated with PFOS levels in the plasma of pregnant women when some other factors (parity, smoking, maternal age, prepregnancy body mass index, and socio-occupational status) were not considered [18]. Kowalczyk et al. [19] described that the total of PFOS and PFOA excretion by way of milk only accounted for approximately 2% among all clearance routes for cows. This value was relatively low, but an investigation estimated the contribution of various food items to daily dietary intake of PFOS and PFOA for the general Norwegian population. As a result, milk and dairy products contributed to 26% of the total PFOS daily intake and 14.5% of the total PFOA daily intake [20]. A similar contribution of 24.5% from milk for the total PFOS dietary intake was presented by a Dutch investigation [13]. It can be concluded that milk and dairy products are potentially important contributors for human exposure to PFOA and PFOS.

The prevalence of PFOA and PFOS in milk and dairy products was largely confirmed in many countries, including China [2,4,8,21,22,23]. However, little is known about the occurrence of PFOA and PFOS in Xinjiang, which has become a large-scale milk production base. Therefore, the present study aimed to characterize the occurrence and distribution of PFOA and PFOS in milk and yogurt collected from Xinjiang. Furthermore, the average daily intake (ADI) of these two substances via milk and yogurt was calculated for a preliminary health risk assessment of the Xinjiang population.

## 2. Materials and Methods

### 2.1. Samples

**Figure 1:**
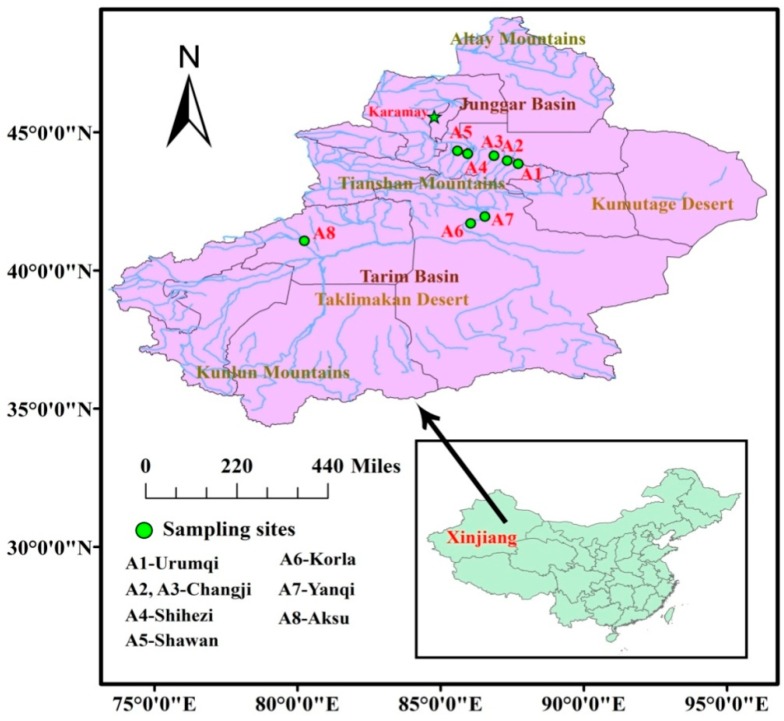
Sampling locations in Xinjiang.

Milk (*n* = 115) and yogurt (*n* = 54) samples from eight producing areas (A1 to A8, Figure 1) were collected between 2014 and 2015 in Xinjiang. These sampling areas represented most of the milk and yogurt production bases in Xinjiang. Milk samples were all from cow milk and were divided into two groups: raw (*n* = 24) and retail milk (*n* = 91). The retail milk and yogurt samples were produced in local areas, and each sample was pooled with five to 10 individuals (randomly purchased from different markets or stores). Raw milk samples (16 from individual cows and eight from bulk storage tanks) were collected from eight farms (A4, Shihezi) and transported on ice. All samples were stored in polypropylene (PP) bottles and shaken vigorously before analysis.

### 2.2. Chemicals and Materials

PFOA (>98%) and PFOS (>98%) were purchased from J&K (Beijing, China) and used as standards. All organic solvents were of high-performance liquid chromatography (HPLC) grade and were provided by Fisher Scientific (Pittsburgh, PA, USA). Ammonium acetate (NH_4_OAc, >97%) and ammonium hydroxide (analytical grade, 25% in water) were also obtained from J&K. Laboratory-produced ultrapure water (>18.2 MΩ/cm) was used throughout the study. Oasis hydrophilic-lipophilic balance (HLB) solid-phase extraction (SPE) cartridges containing 200 mg (6 cc) sorbent with a 30 µm particle size were supplied by Waters (Milford, MA, USA).

### 2.3. Sample Preparation and Instrumental Analysis

The analytical method used in this study was based on the method reported by Wang et al. [21], with some modifications. Briefly, PFOS and PFOA were extracted by ultrasonic extraction with methanol (MeOH) and then purified using solid-phase extraction. First, 3 mL of milk (yogurt) was placed in 50 mL PP centrifuge tubes which had been precleaned with MeOH. The extraction was performed with 15 mL of MeOH by vortex mixing for 30 s, followed by 40 min treatment in an ultrasonic bath. The mixture was centrifuged for 15 min at 6000 r/min, and the supernatant was transferred to another precleaned PP tube. A second extraction was carried out following the steps above, and the supernatants of two fractions were combined and concentrated to a volume of 1 mL under a gentle stream of nitrogen. The concentrated solution was diluted to 100 mL with ultrapure water, and then loaded onto a SPE HLB single-use cartridge (previously activated with 5 mL of MeOH and 10 mL of water). A weak vacuum was used to maintain the flow rate at 1–2 drop/s during the purification process. The cartridge was washed with 5 mL of MeOH/H_2_O (1/20, v/v) and air dried for 30 min under vacuum. Thereafter, PFOA and PFOS were eluted by using 6 mL of 0.4% NH_3_ in MeOH. The obtained eluate was dried under a stream of nitrogen and redissolved with 500 µL of acetonitrile/10 mM NH_4_OAc (45/55, v/v). This extract was filtered using a 0.22 µm nylon mesh filter before analysis.

PFOA and PFOS levels were analyzed by a HPLC tandem mass spectrometry system (HPLC-MS/MS). A 10 μL sample was injected and chromatographed at 40 °C on a Zorbax Eclipse Plus C_18_ 80A analytical column (2.1 mm × 100 mm, 3.5 μm; Agilent Technologies, Santa Clara, CA, USA), which was installed on an Agilent 1200 series HPLC system (Agilent Technologies). 10 mM ammonium acetate aqueous solution/acetonitrile (55/45, v/v) was used as mobile phase at a flow rate of 0.3 mL/min. Liquid chromatography was coupled to an Agilent 6410 triple-quadrupole mass spectrometer operating in negative electrospray ionization mode. Analyses were performed in the multiple reaction monitoring (MRM) mode, and two transitions for PFOS and PFOA were observed. The monitored mass transitions and relative specific parameters are listed in Table 1. Confirmation of analyte identity was based on retention time (RT), in addition to relative intensity ratio of the two mass transitions for each compound.

### 2.4. Quality Control and Quality Assurance

The concentrations of PFOA and PFOS in milk and yogurt samples were determined using the external standard approach. A seven-point standard (0.05 to 100 ng/mL) was prepared for each batch of samples to verify linearity, which was confirmed by *R*^2^ values greater than 0.99 for each compound. Concerning a few lower concentration values are outside the range of the calibration curve, another calibration curve (*R*^2^ > 0.99) in the range 0.005 to 0.05 ng/mL was constructed. The quantitative deviation for PFOA and PFOS at lower levels (0.01, 0.02, and 0.03 ng/mL; 6 times measurement for each levels) using these two different sets of calibration curves was evaluated. However, no significant difference in measurement results by these two calibration curves was observed, which indicated that the calibration curve in this present study could quantify PFOA and PFOS at low levels accurately. A 1000 ng/mL stock solution of each analytes was prepared in a mixture of 55% water (10 mM ammonium acetate) and 45% acetonitrile by volume. Standard mixture of 100 ng/mL were produced by mixing the stock solution of each analytes, adding water/acetonitrile (55/45, v/v). After progressive dilution with water/acetonitrile, the calibration solutions were produced. In addition, the matrix-matched calibration curves were prepared for evaluating matrix effects by comparing the slope of matrix-matched calibration curves and solvent calibration curves. As a result, matrix effects for PFOA and PFOS in milk matrix were not obvious, which was also found by Al-Sheyab et al. [8]. Thus, the matrix-matched calibration curves were not used.

The limits of detection (LOD) were the concentration producing a peak with a signal-to-noise ratio of three, which were 5 and 10 ng/L for PFOA and PFOS, respectively, the limits of quantification (LOQ) were defined as three times the LOD value (Table 1). Recovery test, which was used to examine the accuracy and precision of the analytical method, was performed by analyzing blank milk spiked with standard mixture at three concentrations (0.03, 0.3, and 1 ng/mL). The calculated mean recoveries ranged from 85.4% to 90.1% for PFOA and from 80.3% to 84.3% for PFOS, relative standard deviation (RSD, *n* = 6) for both analytes were lower than 10%. Concentrations measured in samples were recovery-corrected.

To minimize the background contamination, all laboratory vessels were of PP and were rinsed twice with ultrapure water and methanol alternately before use. All the solvents applied in the experiment were analyzed to confirm the absence of PFOA and PFOS. Method blanks were processed along with each batch of samples to monitor the entire pretreatment processes. Neither PFOS nor PFOA were observed above the LOD in blank samples. Good specificity of the method was demonstrated through the analysis of blank milk samples, proving the absence of interfering compounds at retention times of PFOA and PFOS.

### 2.5. Risk Assessment and Statistical Analysis

To elucidate the magnitude of milk and yogurt contribution to human exposure to PFOA and PFOS in Xinjiang, the daily intakes of these two compounds through milk and yogurt should be estimated. However, only retail milk samples were estimated for this purpose because they are the most representative and consumed dairy products among the Xinjiang population. Concentrations below the LOD were considered to be zero. The ADI of PFOS and PFOA was estimated for different groupings (age, area, gender, and race). The ADI of PFOA and PFOS via milk for each group was calculated by multiplying the mean concentrations of analytes in milk by the daily consumption of milk. Dividing the above ADI values by the average body weight of the individuals (assumed 60 kg for an adult [24]) in the group obtained the daily intakes on a body weight basis. Milk consumption data of the Xinjiang population were provided by a recent investigation [25]. Health risk assessment for a general population was estimated by hazard ratios (HRs), which were calculated from dividing the ADI by the reference dose proposed by the European Food Safety Authority with PFOA of 1500 ng/kg/day and PFOS of 150 ng/kg/day. If the HR is >1, health damages are produced.

The SPSS 19.0 statistical software (SSPS Inc., Chicago, IL, USA) was applied to analyze the data. PFOA/PFOS concentrations between the north and south samples were compared using the Mann-Whitney test. Kruskal-Wallis nonparametric tests were performed to reveal the relationship between PFOA/PFOS levels and milk packaging. The correlation between PFOS and PFOA was examined using Spearman’s coefficient.

## 3. Results and Discussion

### 3.1. Concentrations of PFOS and PFOA in Milk and Yogurt

The measured concentrations for PFOS and PFOA, stratified by type, are summarized in Table 2. In retail milk samples, 30 out of 91 samples had detectable levels of PFOA, and 36 of the samples had detectable levels of PFOS. In regard to yogurt, PFOS was present in 26 out of 54 samples, whereas PFOA was detected in 20 cases. The level of PFOA in retail milk ranged from below LOD to 151.8 ng/L (mean = 16.2), which was slightly lower than PFOS (<LOD—172.9 ng/L, mean = 24.5). However, both range and mean concentration for PFOA (<LOD—279.9 ng/L, mean = 22.6) and PFOS (<LOD—200.6 ng/L, mean = 31.8) in yogurt were higher than those in retail milk. Data for PFOS detection rates or mean values were always greater than those of PFOA (Table 2). This finding was similar to several reports [17,20,22,26], probably because of the higher bioaccumulation potential of PFOS in animal biota than that of PFOA. This conjecture is supported by the fact that PFOS was generally the dominating PFAS in the samples of animal origin [20]. Furthermore, PFOS has a higher secretion through milk than PFOA [19,27]. Given the intense ultraviolet radiation in Xinjiang, photochemical degradation of PFOA is likely to happen [28]. This situation can further explain the low PFOA concentrations and detection rates. A recent study on PFOS and PFOA concentrations in human serum was also conducted in Xinjiang [12], and the results showed that PFOS presented a particularly high detection rate (93%) and a low value of 6% for PFOA, thus probably indicating that the environment of Xinjiang region was less contaminated by PFOA than PFOS. This may be related to greater PFOS detection rates and mean values than those of PFOA in milk and yogurt.

PFOS and PFOA concentrations in the present investigation were slightly lower than previous reports [2,8,16,17], probably because the region in this study is predominantly agricultural and pastoral with few industries. Moreover, the region is surrounded by mountains and is far from the ocean. Thus, ocean currents and the long-range atmospheric transport of PFASs are hindered. This situation also causes light pollution in Xinjiang. In the case of raw milk, a few samples turned out to be contaminated with detectable levels of PFOS (three out of 24 samples, 12.5%). It is reported that unprocessed foods were generally free of PFASs and the contamination can occur after the initial production [5,23], which has also been demonstrated by the comparison between raw and retail samples in this study.

Most values below the LOD revealed no widespread contamination of PFOS and PFOA in Xinjiang for milk and yogurt. However, the source of contamination of retail milk in the process of production should be further understood, perhaps by monitoring the entire process line.

### 3.2. Comparison of PFOS and PFOA Concentrations in Samples from Different Areas of Xinjiang

**Figure 2:**
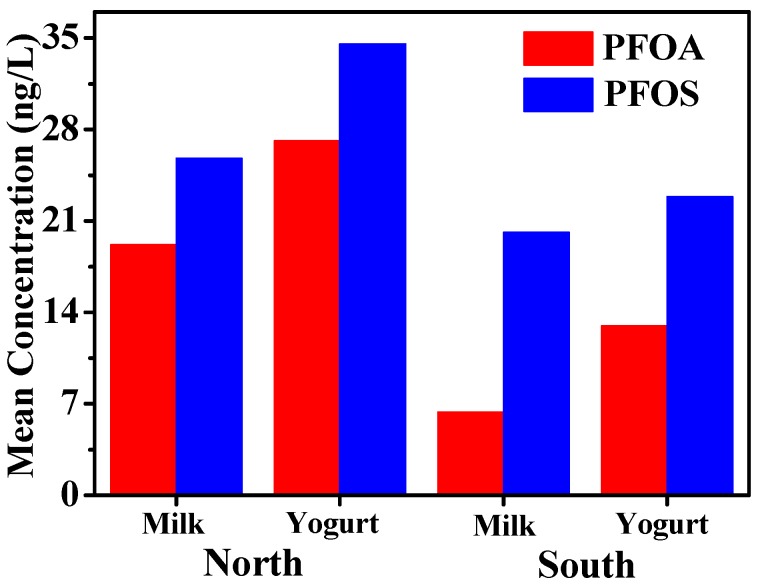
Comparison of mean PFOS and PFOA concentrations in samples from different areas.

Only the retail samples are discussed due to the fact the raw milk samples were collected from eight dairy farms in one area. The eight producing areas of retail samples were divided into two parts (north and south) on the basis of their geographic location within Xinjiang (Figure 1). Urumqi, Changji, Shihezi, and Shawan are cities in northern Xinjiang, whereas Korla, Yanqi, and Aksu belong to southern Xinjiang. PFOA and PFOS contents in milk and yogurt varied with geographic location, as shown in Figure 2 and Table 3. No significant difference (*p* > 0.05) was found in concentrations for each of the analytes between the north and south samples, and relatively high PFOS and PFOA levels were found in the north (Figure 2). On the one hand, wet deposition is considered an important pathway for PFASs from air into soil, water environment, or even food chain [29,30]. Thus, precipitation played an important role in affecting varied contaminant contents between the north and south. Xinjiang belongs to the temperate continental arid climate. Its annual natural precipitation is only 134 mm, and high precipitation was present in northern Xinjiang [31], thus possibly explaining the high levels of PFOS and PFOA in northern Xinjiang. On the other hand, the increased industrial activities in northern Xinjiang were assumed to lead to high PFOA or PFOS exposure in the environment, particularly in Urumqi and Changji, which are the most populated and developed areas in northern Xinjiang. A comparatively high pollution of PFASs is inevitable. Shawan is the least developed among the areas, but presents comparable concentrations of PFOA and PFOS to those of Shihezi, which can be influenced by the neighboring city of Kelamayi. This is a petroleum resource city, and a previous study showed that PFASs were frequently used in the petroleum industry as a clean-up additive [32].

Samples from southern Xinjiang also presented a certain degree of pollution. For PFOS, the mean concentrations were 20.1 and 22.9 ng/L for milk and yogurt, respectively, while for PFOA, the corresponding values were 6.4 and 13.0 ng/L, respectively. This pollution can be attributed to the extensive use of pesticides in southern Xinjiang where the people are mainly engaged in agricultural production. Increased PFOS and PFOA concentrations were found in milk and yogurt from areas with a relatively high level of economic development and industrialization, and this finding agreed well with that found in the north. For instance, in samples from Korla, the concentrations of PFOS and PFOA were even higher than those from northern Xinjiang, except for Urumqi and Changji (Table 3), probably as a consequence of the high gross domestic products (GDP) (rank 2 in Xinjiang) of this region. High concentrations of PFOS in human milk from countries with high GDP have been previously reported [33]. Furthermore, the development of petrochemical industries in the south may increase the PFOA and PFOS load in milk or yogurt. More chemical industrial parks and petrochemical enterprises are emerging in some cities of southern Xinjiang depending on the abundant oil resources provided by the Tarim Oilfield, such as those in Korla and Aksu.

### 3.3. Source Analysis of PFOA and PFOS in Milk and Yogurt

Milk and yogurt may be contaminated by PFOS and PFOA because of cow exposure to air, water, or feed containing perfluorinated chemicals [19,27,34]. A transfer into and an accumulation in terrestrial and aquatic food chains of these compounds have been confirmed by the literature [35,36,37]. Cows, which belong to the upper trophic levels of biota, can bioaccumulate and biomagnificate PFOS and PFOA along the food chains and then biotransfer contaminants from plasma into milk [19,27,38].

**Figure 3:**
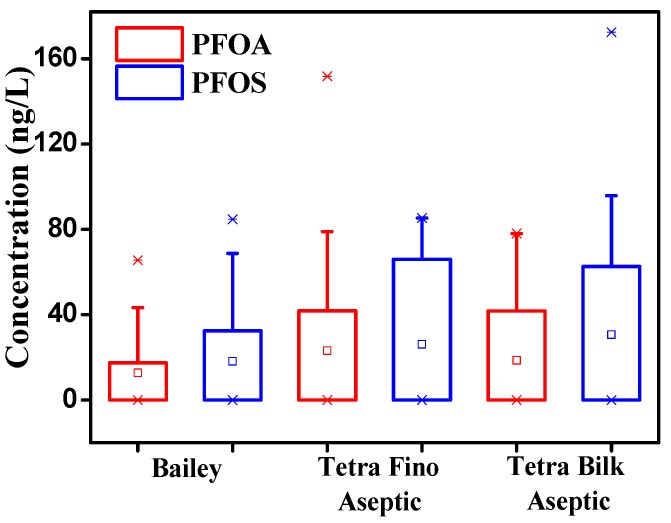
Concentrations (ng/L) of PFOA and PFOS in milk for different packaging materials (Bailey: polyethylene, *n* = 20; Tetra Fino Aseptic: a laminate of paper, polyethylene and aluminum foil, *n* = 20; Tetra Bilk Aseptic: a laminate of paper, polyethylene and aluminum foil, *n* = 20). Note: □, mean; *, maximum and minimum.

In addition, industrial food processing can influence PFOS and PFOA concentrations [5,23]. Enzymatic processes, which occur in the production of yogurt, can increase PFOS and PFOA levels by the enzymatic degradation of their precursors if precursors exist in raw milk [23]. Precursor degradation is regarded as an indirect source of PFOA and PFOS. Parts of their precursors, such as N-ethyl perfluorooctane sulfonamidoethanols and polyfluoroalkyl phosphate diesters, are often used in food packaging materials and have been found in various types of food, including milk and dairy products [7,22,39,40]. The packaging process could be an additional input of PFOA and PFOS either through the direct migration of perfluoroalkyl carboxilic acids or through the migration and subsequent degradation of precursors from packaging materials [23,39]. This migration has been reported in limited types of packaging, such as butter wraps and microwave popcorn bags [23,34]. However, the potential contribution of different types of milk packaging to these two chemicals remains unclear. In this study, PFOA and PFOS levels in milk with different types of packaging were compared, and the results are shown in Figure 3. The concentrations of PFOA and PFOS in the examined milk samples with Tetra Fino Aseptic and Tetra Billk Aseptic were comparable, and both values were higher than those of samples packaged with Bailey. However, Zafeiraki et al. [41] found that neither PFOS nor PFOA were quantified in aluminum foil wrappers, which are commonly used in packaging materials of Tetra Fino Aseptic and Tetra Billk Asepti. Thus, further research is necessary to determine the effects of packaging on PFOA and PFOS concentrations.

### 3.4. Preliminary Health Risk Assessment for the General Xinjiang Population

The daily intakes of PFOA and PFOS through milk were estimated on the basis of milk intake data collected through food frequency questionnaires in a 2010 Xinjiang survey [25]. Participants for the survey were selected from among 3000 adults, including five groups (age, gender, area1, area2, and race). In-person interviews and questionnaires were administered, and participants provided detailed information regarding their diet over the past one year. Individual food items were quantified into grams per day, and daily consumption values of milk (DC_milk_, g/day) for age groups were 55 (18–29 years), 65 (30–39 years), 74 (40–49 years), 75 (50–59 years), 93 (60–69 years) and 113 (≥70 years), respectively. DC_milk_ of other groups is shown in Table 4.

**Figure 4:**
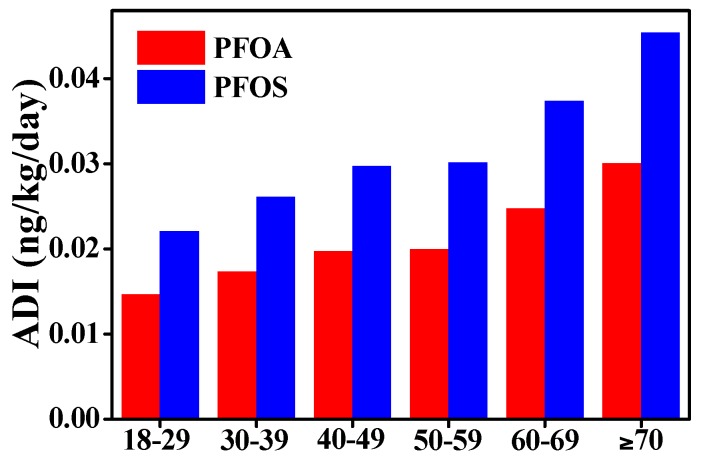
Average daily intake of PFOA and PFOS from milk and dairy products on the basis of body weight (60 kg) by the adults of Xinjiang.

**Figure 5:**
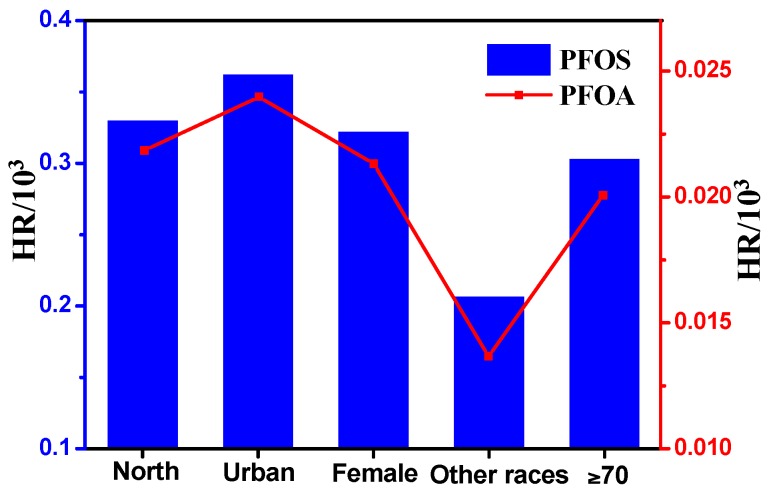
Health risk comparison of five population groups via intake of PFOA and PFOS from milk and dairy products.

The ADI of PFOS and PFOA for the six age groups is presented in Figure 4. The ADI_PFOS_ for the general Xinjiang adult population (≥18 years old) was 0.0318 ng/kg/day, which exceeded ADI_PFOA_ (0.0211 ng/kg/day). The calculated intakes in the present study are similar to the reports of Wang et al. [21] and Ericson et al. [42], but lower than those reported in a Norwegian study [20]. The different estimated intakes can be related to the differences in background levels of contaminants for milk, consumption data of various regions, and data treatment on non-detects. For both PFOS and PFOA, the estimated daily intake increased with increasing age, and the highest intake was found in the elderly aged over 70 years old (PFOA: 0.0301 ng/kg/day, PFOS: 0.0454 ng/kg/day), whereas an opposite trend was reported by Haug et al. [20]. This phenomenon can be explained by the extremely large consumption of milk (113 g/day) for the elderly in the present study. The ADI values of two compounds for various groupings (area, gender, and race) are presented in Table 4. The food survey of Xinjiang reported a significant difference (*p* < 0.05) in the daily consumption of milk among each group, except for gender. Therefore, the ADI values varied depending on the consumption patterns of each group. The HR values of PFOA and PFOS was only calculated for five population groups (north, urban, female, other races, and ≥70 years) because they owned the highest ADI of PFOA and PFOS in their respective groups (Figure 4 and Table 4) and the result was shown in Figure 5. The computed HRs (PFOA_max_: 0.024 × 10^−3^, PFOS_max_: 0.362 × 10^−3^) will increase if yogurt was involved in estimation, but the values will still be far less than one for all population groups. This finding indicates that the risk related to the intake of PFOS and PFOA through milk and yogurt is low for the Xinjiang population.

## 4. Conclusions

In summary, this study is the first large-scale survey to assess the presence of PFOA and PFOS in milk and yogurt from Xinjiang. No widespread contamination of PFOS and PFOA in milk and yogurt was found in Xinjiang because only a fraction of the analyzed samples contained detectable concentrations of the two contaminants. The levels in this study were lower than those of previous reports. PFOS presents relatively higher concentrations than PFOA in both milk and yogurt, which could be attributed to the fact that PFOS has a higher bioaccumulation potential in animals than PFOA. Relatively high levels of PFOS and PFOA were found in the north of Xinjiang, and this finding may result from the high precipitation and developed industries. Furthermore, the HR values indicated that prevalent concentrations of PFOS and PFOA in milk and yogurt are unlikely to generate an immediate risk to the Xinjiang population. However, precursor degradation is a potential indirect route of human exposure to PFOA and PFOS. Therefore, further research should be conducted to investigate the contents of their precursors in milk and dairy products, considering that PFOA or PFOS load in humans can be increased by precursor biotransformation or metabolism in the body. Moreover, serum concentrations of PFOS and PFOA in the local population should be monitored to enhance our knowledge about the total dosage of these two contaminants with regard to various exposure sources by using pharmacokinetics models.

## Figures and Tables

**Table 1:** MS/MS MRM parameters and calculated LOD and LOQ for PFOS and PFOA.

Analyte	Mass Transition ^a^	Fragment (V)	CE (V)	RT (min)	LOD (ng/L)	LOQ (ng/L)
PFOS	**499.0→99.0**	160	55	6.7	10	30
	499.0→80.0	160	72			
PFOA	**412.8→368.8**	70	4	2.0	5	15
	412.8→168.9	70	16			

**^a^** Transitions used for quantification are shown in bold; CE: collision energy; RT: retention time; LOD: the limits of detection; LOQ: the limits of quantification; MRM: multiple reaction monitoring; PFOS: perfluorooctane sulfonate; PFOA: perfluorooctanoic acid.

**Table 2:** Concentrations (ng/L) of PFOA and PFOS in milk and yogurt samples.

Type	PFOA ^1^	PFOS ^1^
Retail milk, *n* = 91		
Range	<5.0–151.8	<10.0–172.9
Mean	16.2	24.5
Frequency values > LOD (%)	33.0	39.6
Raw milk, *n* = 24		
Range		<10.0–25.1
Mean		2.2
Frequency values > LOD (%)		12.5
Yogurt, *n* = 54		
Range	<5.0–279.9	<10.0–200.6
Mean	22.6	31.8
Frequency values > LOD (%)	37.0	48.1

**^1^** No correlation was found between the measured concentrations of the two compounds in each sample.

**Table 3:** Concentrations (ng/L) of PFOA and PFOS in different areas.

Areas	Type	Number	PFOA			PFOS		
			Max	Mean	Frequency	Max	Mean	Frequency
Urumqi	^a^M	36	151.8	27.1	47.2	172.9	35.7	50.0
	^b^Y	10	279.9	39.1	50.0	200.6	48.2	60.0
^1^Changji	M	10	79.0	16.6	40.0	95.8	20.8	40.0
	Y	14	199.9	27.7	42.9	117.3	36.3	50.0
Shihezi	M	12	65.6	9.3	25.0	74.8	13.7	25.0
	Y	6	54.0	15.7	33.3	91.4	24.0	33.3
Shawan	M	12	56.8	7.5	16.7	53.8	12.1	25.0
	Y	6	77.0	12.8	16.7	84.3	22.2	33.3
Korla	M	7	42.3	16.9	42.9	88.2	32.0	57.1
	Y	6	94.9	23.1	50.0	152.7	34.7	50.0
Yanqi	M	7	<LOD	<LOD	0	49.8	13.6	28.6
	Y	6	28.4	4.7	16.7	45.0	14.9	33.3
Aksu	M	7	15.1	2.2	14.3	81.5	14.7	28.6
	Y	6	66.7	11.1	16.7	88.1	19.0	50.0

**^a^**M: milk, **^b^**Y: yogurt. **^1^**Changji: A2, A3.

**Table 4:** Average daily intake of PFOS and PFOA for various groups in terms of area, gender, and race.

Group		DC_milk_ g/day	ADI ng/kg/day ^a^	
			PFOA	PFOS
Area1	North	123	0.0328	0.0495
	South	15	0.0040	0.0060
Area2	Urban	135	0.0360	0.0543
	Rural	54	0.0144	0.0217
Gender	Male	71	0.0190	0.0286
	Female	77	0.0205	0.0310
Race	Han Chinese	118	0.0314	0.0474
	Uighur	32	0.0086	0.0129
	Others	120	0.0320	0.0483

**^a^**Calculated using a body weight of 60 kg. DC_milk_: daily consumption values of milk.
